# Immunotherapy Is Associated with a Survival Benefit in Patients Receiving Chemotherapy for Metastatic Pancreatic Cancer

**DOI:** 10.1089/pancan.2021.0003

**Published:** 2021-04-12

**Authors:** Jonathan J. Hue, Katherine Bingmer, Kavin Sugumar, Sarah C. Markt, Luke D. Rothermel, Jeffrey M. Hardacre, John B. Ammori, Jordan M. Winter, Lee M. Ocuin

**Affiliations:** ^1^ Division of Surgical Oncology, Department of Surgery, University Hospitals Cleveland Medical Center, Cleveland, Ohio, USA.; ^2^ Department of Population and Quantitative Health Sciences, Case Western Reserve University School of Medicine, Cleveland, Ohio, USA.

**Keywords:** pancreatic cancer, immunotherapy, chemotherapy, stage IV disease

## Abstract

**Background:** Immunotherapy (IT) has led to improved survival in several common cancers but success in pancreatic ductal adenocarcinoma (PDAC) has been limited. We analyzed if combination IT–chemotherapy (IT-CT) is associated with improved survival compared with chemotherapy alone (CT) in patients with metastatic PDAC.

**Methods:** The National Cancer Database (2004–2016) was queried for patients who were diagnosed with metastatic PDAC. Patients were categorized by treatment group: CT only and IT-CT. Patients were excluded if they received radiation or a surgical procedure. The primary outcome was overall survival.

**Results:** A total of 59,289 patients were identified, of whom 58,947 (99.4%) received CT and 342 (0.6%) received IT-CT. The IT-CT group was younger, had fewer comorbidities, and was more often treated at an academic center. The utilization of multiagent CT was similar between the groups. Median survival of patients treated with IT-CT was longer than CT alone (7.9 months vs. 6.3 months, *p* = 0.005). On multivariable analysis, receipt of IT-CT was associated with a survival advantage as compared with CT (hazard ratio = 0.86, 95% confidence intervals 0.76–0.97) when adjusting for demographics and type of CT regimen.

**Conclusion:** In patients with metastatic PDAC, it appears that combination IT-CT may perhaps be associated with a survival advantage compared with CT alone.

## Introduction

Multiagent chemotherapy (CT) remains the backbone of systemic treatment for patients with pancreatic ductal adenocarcinoma (PDAC).^1^ FOLFIRINOX (5-fluorouracil, leucovorin, irinotecan, oxaliplatin), gemcitabine with nab-paclitaxel, and gemcitabine with capecitabine have demonstrated a survival benefit over gemcitabine monotherapy in patients with metastatic PDAC.^2–4^ Despite modest advances in the effectiveness of chemotherapeutics, further combinations of existing chemotherapies are unlikely due to compounding side effect profiles. Multiagent CT regimens are associated with severe toxicities, which have been reported in the majority of patients receiving FOLFIRINOX.^2,5^ Alternative treatment strategies are needed. There has been improvement in overall survival of other solid tumor malignancies over the past decade, such as melanoma, renal cell carcinoma, lung cancer, and bladder cancer, with the introduction of therapy targeting various immune system checkpoints.^6–9^ Immunotherapy (IT) aims to boost a patient's immune system to enhance tumor recognition and destruction.^10–14^ Therapies aimed at modulating the immune response to cancer (i.e., cancer vaccines, oncolytic viruses, etc.) have been under investigation for decades,^15–18^ but current immune checkpoint inhibitors were first approved in 2011 after demonstrating a survival benefit among patients with metastatic melanoma.^19^ Unfortunately, no such success has been realized in the treatment of PDAC to date.^20–26^

A previous retrospective analysis of patients with PDAC who underwent a pancreatectomy in the National Cancer Database (NCDB) demonstrated an association between improved overall survival and adjuvant IT-CT as compared with adjuvant CT alone.^27^ To our knowledge, there has not been a report on the impact of IT in metastatic PDAC patients using a large administrative database. The primary aim of this study was to use the NCDB to determine if the addition of IT to CT was associated with a survival advantage in patients with metastatic PDAC. In addition, we aimed to identify potential disparities in receipt of IT based on sociodemographic characteristics.

## Materials and Methods

### Institutional assurances

This project was exempt from approval by the Institutional Review Board at our institution under Federal Regulation 45 CFR 46.101 (b).

### Data source

The NCDB is a nationwide database, jointly maintained by the American College of Surgeons and the American Cancer Society. This database receives patient information from Commission on Cancer-accredited hospitals across the United States and contains deidentified information on ∼70% of all new malignancies diagnosed in the United States each year. The accuracy of the data reported, statistical analyses performed, and conclusions drawn are not monitored nor verified by the American College of Surgeons or American Cancer Society. The NCDB Participant User File data dictionary contains definitions of variables used in this study.^28^

### Patient cohort

**FIG. 1.**
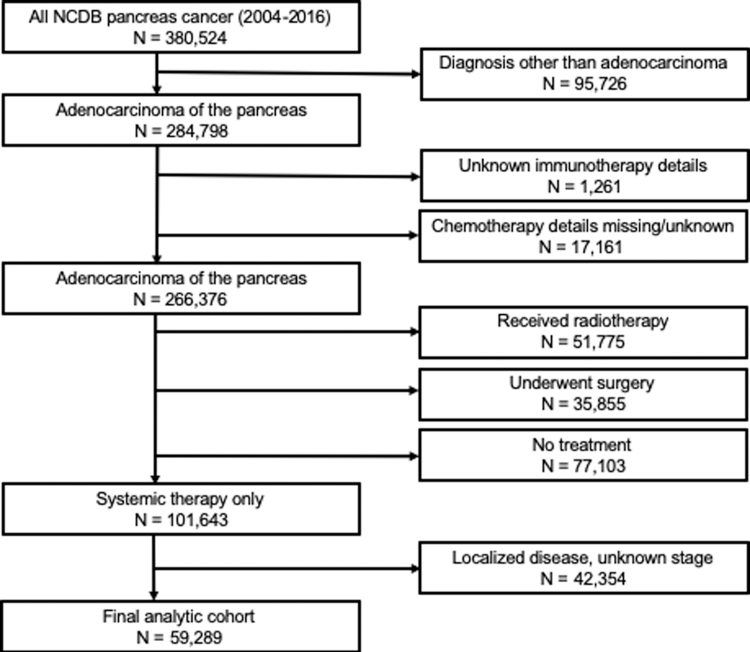
Consort diagram of study population.

Adult patients diagnosed with PDAC were identified between 2004 and 2016, using *International Classification for Diseases in Oncology* (third edition) codes 8140 and 8500. Only patients with metastatic (stage IV) disease at diagnosis were included. Patients were excluded from the analysis if they underwent a surgical procedure, if they received radiotherapy, if the type of CT administered (single-agent or multiagent) was not recorded, or if data on IT administration was missing (consort diagram, Fig. 1). For this analysis, we utilized data on sociodemographic (age, sex, race, insurance status, education quartile, income quartile, Charlson–Deyo comorbidity index) and clinical characteristics, facility type (academic, community, comprehensive community, integrated), and treatment information.

### Exposure

Patients with PDAC were categorized into two treatment groups: those who received single- or multiagent CT alone or IT and CT (IT-CT). Within the NCDB, IT is defined as biological or chemical agents that alter the immune system or change the host's response to tumor cells.^28^

### Outcomes

The primary outcome in this study was overall survival, defined as the number of months from the patient's date of diagnosis to either their date of death or last follow-up. Cancer-specific outcomes were not evaluated because the NCDB does not capture data pertaining to progression or cause of death.

### Statistical analyses

Clinical and demographic data were compared between the treatment groups. The Wilcoxon rank-sum test was used to compare continuous dependent variables. Pearson's chi-squared test was used to compare categorical variables. Multivariable logistic regression was used to determine the association between IT usage and demographic characteristics and clinical variables. Median survival was analyzed using the Kaplan–Meier estimate and compared by the log-rank test. Multivariable Cox proportional hazards regression models were used to calculate hazard ratios (HRs) and 95% confidence intervals (95% CI) for the association between the treatment groups and survival, adjusting for sex, age, race (white, other), type of hospital (academic, other), insurance status (private, other), and Charlson–Deyo comorbidity score (0, ≥1). The NCDB does not include survival data for patients diagnosed in 2016, thus these patients were not included in survival analyses. StataSE v16.0 (Statacorp LLC, College Station, TX) was used for statistical analyses. A *p*-value <0.05 was used to indicate statistical significance.

## Results

### Demographic and clinical data

A total of 59,289 patients were identified, of whom 58,947 (99.4%) received CT and 342 (0.6%) received IT-CT (Table 1). Patients who received IT-CT were younger (64 years vs. 66 years, *p* = 0.002), more likely to be white, and more likely to have private insurance as compared with those who received CT. Patients who received IT-CT had fewer comorbidities as compared with patients who received CT (Charlson–Deyo score = 0: 76.3% vs. 68.9%, *p* = 0.007). The majority of patients in the IT-CT group were treated at academic hospitals (56.6% IT-CT vs. 39.7% CT, *p* < 0.001). Usage of multiagent CT was similar between the groups.

**Table 1.** Summary of demographic and clinical data among patients with metastatic pancreatic ductal adenocarcinoma (National Cancer Database, 2004–2016)

Characteristic	IT-CT (n* = 342), *n (%)	CT (n* = 58,947), *n (%)	P
Age, years, median (range)	64 (21–86)	66 (18–90)	**0.002**
Male	187 (54.7)	32,058 (54.4)	0.913
Race/Ethnicity			**0.008**
White	278 (81.3)	44,770 (75.9)	
Black	21 (6.1)	6960 (11.8)	
Hispanic	18 (5.3)	2465 (4.2)	
Other	25 (7.3)	4752 (8.1)	
Primary insurance payor			**0.001**
None	4 (1.2)	1744 (3.0)	
Private	168 (49.1)	23,236 (39.4)	
Medicare	140 (40.9)	29,183 (49.5)	
Medicaid, other	30 (8.8)	4784 (8.1)	
Education: <6.3% of population w/o high school diploma	113 (33.5)	15,862 (27.3)	**0.031**
Income: ≥ $63,333 annual household income	151 (45.1)	22,149 (38.2)	**0.001**
Charlson–Deyo score = 0	261 (76.3)	40,639 (68.9)	**0.007**
Facility type			**<0.001**
Community	25 (7.5)	4744 (8.1)	
Comprehensive community	97 (29.0)	22,899 (39.3)	
Academic/research	189 (56.6)	23,126 (39.7)	
Integrated network	23 (6.9)	7537 (12.9)	
Distance to hospital, miles, median (IQR)	17.8 (6.7–46.6)	10.1 (4.5–24.2)	**<0.001**
Tumor size, mm, median (IQR)	40 (30–52)	38 (30–51)	0.520
Multiagent CT	228 (66.7)	37,043 (62.8)	0.144
Single-agent CT	114 (33.3)	21,904 (37.2)	

Bold values indicate statistical significance.

CT, chemotherapy; IQR, interquartile range; IT, immunotherapy.

### Factors associated with utilization of IT

Multivariable logistic regression was used to determine what factors were associated with the administration of IT (Table 2). Advanced age and greater comorbidity score were associated with a decreased likelihood of receiving IT. Treatment at an academic facility was associated with an increased likelihood of receiving IT (odds ratio = 1.95, 95% CI 1.58–2.41). There was no association between receipt of IT and sex, race, or insurance payor.

**Table 2.** Multivariable logistic regression analyzing utilization of immunotherapy among patients with metastatic pancreatic ductal adenocarcinoma (National Cancer Database, 2004–2016)

	OR	95% CI	p
Age	0.99	0.98–1.00	**0.046**
Female vs. male	1.01	0.82–1.25	0.929
White vs. other race	1.30	0.99–1.71	0.055
Private insurance vs. other	1.23	0.96–1.56	0.100
Top 2 income quartiles vs. bottom	0.86	0.66–1.12	0.277
Top 2 education quartiles vs. bottom	1.34	1.03–1.74	**0.031**
Academic facility vs. other	1.95	1.58–2.41	**<0.001**
Charlson–Deyo score ≥1 vs. 0	0.77	0.60–0.99	**0.040**

Bold values indicate statistical significance.

95% CI, 95% confidence intervals; OR, odds ratio.

### Survival analyses

**FIG. 2.**
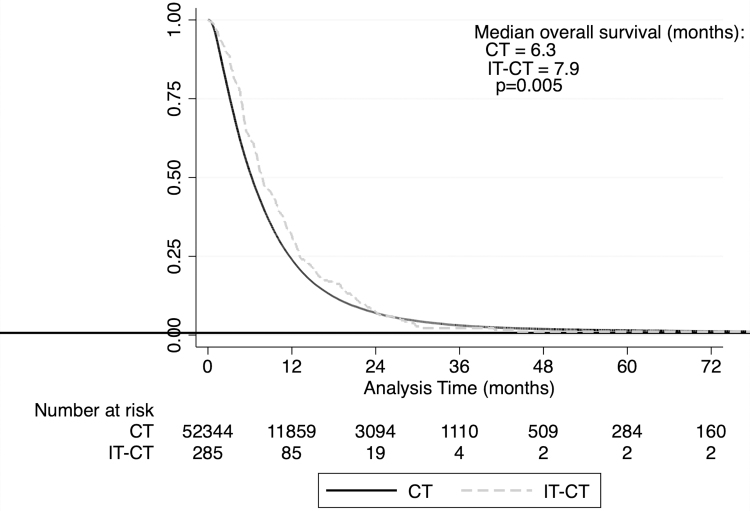
Kaplan–Meier estimates of survival by treatment group (NCDB, 2004–2015). NCDB, National Cancer Database.

Patients who received combined IT-CT had longer median overall survival than patients treated with CT alone (7.9 months vs. 6.3 months, *p* = 0.005; Table 3 and Fig. 2). The 2-year survival rates were similar (IT-CT: 7.32%, CT: 7.01%). Of note, there was an additional group of patients who received IT without CT. This group (*n* = 17) had a median survival of 5.2 months.

**Table 3.** Summary of median survival among patients with metastatic pancreatic ductal adenocarcinoma stratified by treatment group (National Cancer Database, 2004–2015)

Treatment	n	Median	IQR	p
IT-CT	268	7.9	4.8–13.1	0.005
CT	52,344	6.3	3.2–11.7	
IT only	17	5.2	2.7–15.5	—

**FIG. 3.**
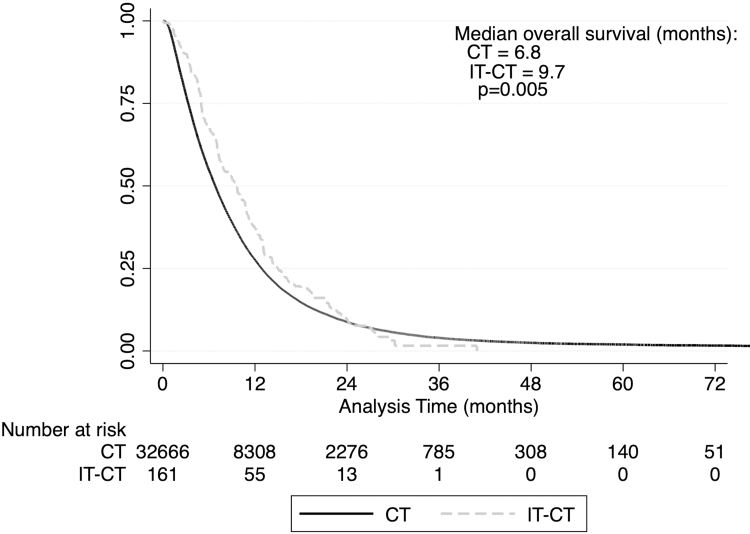
Kaplan–Meier estimates of survival by treatment group (NCDB, 2010–2015).

Additionally, we performed a subgroup analysis for patients diagnosed in 2010 or later as both FOLFIRINOX and gemcitabine/nab-paclitaxel were introduced around that time.^2,3^ We found that the median survival of patients who received IT-CT (*n* = 161) increased to 9.7 months and was longer than those who received CT only (*n* = 32,666, 6.8 months, *p* = 0.017, Fig. 3).

A multivariable Cox regression analysis examining overall survival in the treatment groups is shown in Table 4. IT-CT was associated with a survival advantage relative to CT alone (HR = 0.86, 95% CI 0.76–0.97). Patients with advanced age, those treated at nonacademic facilities, and those with a greater Charlson–Deyo score also had an associated survival disadvantage. Those who received multiagent CT had an associated survival benefit as compared with those who received single-agent CT (HR = 0.65, 95% CI 0.64–0.66).

**Table 4.** Multivariable Cox proportional hazards regression analyzing overall survival between treatment groups (National Cancer Database, 2004–2015)

	HR	95% CI	p
IT-CT vs. CT	0.86	0.76–0.97	0.015
Age	1.01	1.00–1.01	<0.001
Female vs. male	0.93	0.91–0.95	<0.001
Academic facility vs. other	0.83	0.82–0.85	<0.001
Private insurance vs. other	0.94	0.92–0.96	<0.001
Charlson–Deyo score ≥1 vs. 0	1.13	1.11–1.15	<0.001
Multiagent CT vs. single-agent CT	0.65	0.64–0.66	<0.001

HR, hazard ratio.

## Discussion

In recent years, IT has revolutionized the field of oncology. Several malignancies in which IT has improved survival, such as melanoma, lung, and renal cell carcinoma, are well vascularized, highly immunogenic, have a high mutational burden, and have a microenvironment conducive to immune cell survival and function.^29,30^ Despite encouraging results in other malignancies, IT has not demonstrated efficacy in the majority of PDAC patients. More recently, olaparib (a PARP inhibitor) was approved for utilization in selected patients with metastatic pancreatic cancer. There was no difference in median survival as compared with placebo (18.9 months vs. 18.1 months), but olaparib was associated with a greater progression-free survival (7.4 months vs. 3.8 months).^31^ Of note, olaparib was only approved for patients with *BRCA* (breast cancer gene)-mutated pancreatic cancer, which accounts for just 5% of cases.^32^ The median survival of both arms in the aforementioned study was greater than the current analysis. To be included in this trial, patients had to complete 16 weeks of CT, likely preventing enrollment of patients with poor performance status or rapidly progressive disease. Therefore, results of the olaparib trial are likely not broadly generalizable to the majority of patients with metastatic PDAC.

In our study, we report that the addition of IT to CT was associated with a survival advantage in patients with metastatic PDAC. Median overall survival was ∼1.6 months longer in the IT-CT group. This is concordant with a recent report which demonstrated that the combination of IT and CT in the adjuvant setting was associated with improved survival compared with adjuvant CT alone (5-year overall survival: 30.3% vs. 20.6%).^27^ The associated survival advantage among patients who received adjuvant IT-CT as compared with adjuvant CT was greater in magnitude relative to the findings in our study. We hypothesize that this discrepancy may be due to the effectiveness of treatments based on disease burden. For example, when compared with single-agent gemcitabine, FOLFIRINOX leads to a 19.4-month survival advantage in the adjuvant setting, but just a 4.3-month advantage in metastatic PDAC.^2,5^ It is possible that our current findings, as well as those reported in the adjuvant setting, are false-positive results, attributable to randomness in a relatively small sample size or unappreciated confounders.

Numerous immune targets have been studied in PDAC. A phase II trial in patients with metastatic PDAC who failed first-line CT received durvalumab, a monoclonal antibody targeting PD-L1 (programmed death-ligand 1); however, this trial was terminated due to a response rate of 0%.^23^ Cytotoxic T lymphocyte-associated protein-4 (CTLA-4), another immune checkpoint, has been shown to downregulate immune responses. A single-arm clinical trial, which combined ipilimumab (a CTLA-4 antagonist), nivolumab (a PD-1 [programmed cell death protein 1] antagonist), and radiation in patients with metastatic PDAC demonstrated an objective response rate of just 14% and a progression-free survival of 76 days.^24^ A recent phase I trial examining the safety of nivolumab, nab-paclitaxel, and gemcitabine in patients with advanced PDAC did not show a survival benefit as compared with historic survival data without nivolumab.^33^

Deficiencies in mismatch repair with subsequent microsatellite instability (MSI) are often found in cancers and cause numerous genetic mutations, activating a patient's antitumor immune response.^34^ A pivotal trial administered pembrolizumab (a PD-1 antagonist) to patients with metastatic cancer and demonstrated a disease control rate of 75% and a reported objective response rate of 62% in the limited subset of patients with pancreatic cancer (*n* = 8).^35^ A follow-up trial, which also administered pembrolizumab to MSI-high/mismatch repair-deficient advanced cancers, reported an objective response rate in tumors of pancreatic origin of just 18% with progression-free and overall survival of 2 and 4 months, respectively.^22^ Importantly, only 1% to 2% of PDAC tumors are MSI-high and/or mismatch repair deficient.^36^ It should be emphasized that the impact of IT on PDAC with MSI remains unproven, and the incidence of these tumors is extremely rare. One potential explanation for the discrepancy in effectiveness of IT relative to other cancers is due to the austere tumor microenvironment of PDAC, which includes a dense stromal compartment, low nutrient concentrations, and relative hypoxia.^20,37^ Patients' immune cells, which immunotherapies rely upon, are likely less effective under these harsh conditions, since they are not well adapted to survive in this harsh environment.^37,38^ A recent study by our group demonstrated that among all active phase III trials in the United States, 37% targeted the immune system.^39^ While our data show that some patients benefit from IT, the 2-year survival rate was just 7%. Thus, ongoing research efforts may want to investigate other potential targets.

Our study also highlights disparities in utilization of IT. We found that advanced age and greater comorbidity index were associated with a decreased utilization of IT. Higher level education and receiving treatment at an academic center were also associated with increased IT utilization. Disparities in cancer are well studied and previous reports have shown variations in therapies administered and survival based on race and socioeconomic status.^40,41^ Studies have recommended multiple strategies, including insurance reform, improved access to quality care with community outreach, improved access to novel therapies, increased emphasis on prevention and screening, and diversification of employees in the health care field, to reduce disparities in cancer care.^42–44^ Previous reports identified that Black and Asian patients, those who live alone, were unmarried or did not have children, had government-issued insurance, or had issues performing their activities of daily living were more likely to decline all treatment.^45,46^

Although our study includes data on a large number of PDAC patients, there are limitations. Importantly, the use of IT is not common, therefore the IT-CT treatment group is quite small in comparison to those who received CT alone. Those who received IT likely represent a highly select patient cohort, which may not be generalizable to all patients with metastatic PDAC. We attempted to control for this by including appropriate variables (age, insurance payor, treating facility type, and Charlson–Deyo comorbidity index) in our multivariable models. Based on limitations inherent to NCDB studies, including the lack of granularity of the data, we cannot comment on specific treatment details, such as the type of CT or IT (oncolytic virus, cancer vaccine, immune checkpoint inhibitors) received, whether the IT was part of a clinical trial, how many cycles of CT were completed, the genetic make-up of a patient's tumor (BRCA mutation, MSI status, etc.), and why IT was administered (progression of disease, patient desire, clinical trial, etc.). Also, the NCDB only includes data on the first 6 months of treatment, therefore a subset of patients in the CT group may have received IT later in their treatment course. Details of a patient's performance status are not recorded in the NCDB, thus Charlson–Deyo scores were used as a surrogate. The NCDB only receives data from the Commission on Cancer-accredited hospitals and only captures 70% of cancer diagnoses in the United States. Therefore, these data cannot truly be generalized to all hospital systems and patients. Despite these limitations, our study reports a thorough analysis of the utilization of IT in patients with metastatic PDAC and demonstrates there may be a subset of patients who respond to IT.

## Conclusion

In patients with metastatic PDAC, the addition of IT to CT was associated with improved median survival but a similar 2-year survival rate. Future research is needed to identify strategies that both stimulate entry of antitumor immune cells into the PDAC microenvironment and increase the antitumor capabilities of these cells.

## Preprint

An earlier draft of this article was posted at Research Square (DOI: 10.21203/rs.3.rs-121110/v1).

## Abbreviations Used

BRCA: breast cancer gene
95% CI: 95% confidence interval
CT: chemotherapy
CTLA-4: cytotoxic T lymphocyte-associated protein-4
FOLFIRINOX: 5-fluorouracil, leucovorin, irinotecan, oxaliplatin
HR: hazard ratio
IQR: interquartile range
IT: immunotherapy
MSI: microsatellite instability
NCDB: National Cancer Database
OR: odds ratio
PARP: poly (adenosine diphosphate–ribose) polymerase
PD-1: programmed cell death protein 1
PDAC: pancreatic ductal adenocarcinoma
PD-L1: programmed death-ligand 1

## References

[1] NCCN guidelines version 1. 2019 pancreatic adenocarcinoma. National Comprehensive Cancer Network. Available at https://www.nccn.org/professionals/physician_gls/PDF/pancreatic.pdf Accessed 39, 2020

[2] Conroy T, Desseigne F, Ychou M, et al. FOLFIRINOX versus gemcitabine for metastatic pancreatic cancer. N Engl J Med. 2011;364:1817–1825 21561347 10.1056/NEJMoa1011923

[3] Von Hoff DD, Ervin T, Arena FP, et al. Increased survival in pancreatic cancer with nab-paclitaxel plus gemcitabine. N Engl J Med. 2013;369:1691–1703 24131140 10.1056/NEJMoa1304369 PMC4631139

[4] Cunningham D, Chau I, Stocken DD, et al. Phase III randomized comparison of gemcitabine versus gemcitabine plus capecitabine in patients with advanced pancreatic cancer. J Clin Oncol. 2009;27:5513–5518 19858379 10.1200/JCO.2009.24.2446

[5] Conroy T, Hammel P, Hebbar M, et al. FOLFIRINOX or gemcitabine as adjuvant therapy for pancreatic cancer. N Engl J Med. 2018;379:2395–2406 30575490 10.1056/NEJMoa1809775

[6] Powles T, Eder JP, Fine GD, et al. MPDL3280A (anti-PD-L1) treatment leads to clinical activity in metastatic bladder cancer. Nature. 2014;515:558–564 25428503 10.1038/nature13904

[7] Choueiri TK, Xie W, Kollmannsberger C, et al. The impact of cytoreductive nephrectomy on survival of patients with metastatic renal cell carcinoma receiving vascular endothelial growth factor targeted therapy. J Urol. 2011;185:60–66 21074201 10.1016/j.juro.2010.09.012

[8] Dobry AS, Zogg CK, Hodi FS, et al. Management of metastatic melanoma: improved survival in a national cohort following the approvals of checkpoint blockade immunotherapies and targeted therapies. Cancer Immunol Immunother. 2018;67:1833–1844 30191256 10.1007/s00262-018-2241-x PMC6249064

[9] Rizvi NA, Mazières J, Planchard D, et al. Activity and safety of nivolumab, an anti-PD-1 immune checkpoint inhibitor, for patients with advanced, refractory squamous non-small-cell lung cancer (CheckMate 063): a phase 2, single-arm trial. Lancet Oncol. 2015;16:257–265 25704439 10.1016/S1470-2045(15)70054-9 PMC5726228

[10] Kunk PR, Bauer TW, Slingluff CL, et al. From bench to bedside a comprehensive review of pancreatic cancer immunotherapy. J Immunother Cancer. 2016;41–12 10.1186/s40425-016-0119-z PMC4791889 26981244

[11] Immunotherapy to treat cancer. National Cancer Institute. Available at https://www.cancer.gov/about-cancer/treatment/types/immunotherapy Accessed 39, 2020

[12] Manji GA, Olive KP, Saenger YM, et al. Current and emerging therapies in metastatic pancreatic cancer. Clin Cancer Res. 2017;23:1670–1678 28373365 10.1158/1078-0432.CCR-16-2319

[13] Torphy RJ, Zhu Y, Schulick RD. Immunotherapy for pancreatic cancer: barriers and breakthroughs. Ann Gastroenterol Surg. 2018;2:274–281 30003190 10.1002/ags3.12176 PMC6036358

[14] Guo S, Contratto M, Miller G, et al. Immunotherapy in pancreatic cancer: unleash its potential through novel combinations. World J Clin Oncol. 2017;8:230–240 28638792 10.5306/wjco.v8.i3.230 PMC5465012

[15] Jaffee EM, Hruban RH, Biedrzycki B, et al. Novel allogeneic granulocyte-macrophage colony-stimulating factor-secreting tumor vaccine for pancreatic cancer: a phase I trial of safety and immune activation. J Clin Oncol. 2001;19:145–156 11134207 10.1200/JCO.2001.19.1.145

[16] Laheru D, Lutz E, Burke J, et al. Allogeneic granulocyte macrophage colony-stimulating factor-secreting tumor immunotherapy alone or in sequence with cyclophosphamide for metastatic pancreatic cancer: a pilot study of safety, feasibility, and immune activation. Clin Cancer Res. 2008;14:1455–1463 18316569 10.1158/1078-0432.CCR-07-0371 PMC2879140

[17] Gjertsen MK, Buanes T, Rosseland AR, et al. Intradermal ras peptide vaccination with granulocyte-macrophage colony-stimulating factor as adjuvant: clinical and immunological responses in patients with pancreatic adenocarcinoma. Int J Cancer. 2001;92:441–450 11291084 10.1002/ijc.1205

[18] Hecht JR, Bedford R, Abbruzzese JL, et al. A phase I/II trial of intratumoral endoscopic ultrasound injection of ONYX-015 with intravenous gemcitabine in unresectable pancreatic carcinoma. Clin Cancer Res. 2003;9:555–561 12576418

[19] Hodi FS, O'Day SJ, McDermott DF, et al. Improved survival with ipilimumab in patients with metastatic melanoma. N Engl J Med. 2010;363:711–723 20525992 10.1056/NEJMoa1003466 PMC3549297

[20] Looi CK, Chung FFL, Leong CO, et al. Therapeutic challenges and current immunomodulatory strategies in targeting the immunosuppressive pancreatic tumor microenvironment. J Exp Clin Cancer Res. 2019;38:1–23 30987642 10.1186/s13046-019-1153-8 PMC6463646

[21] Hilmi M, Bartholin L, Neuzillet C. Immune therapies in pancreatic ductal adenocarcinoma: where are we now? World J Gastroenterol. 2018;24:2137–2151 29853732 10.3748/wjg.v24.i20.2137 PMC5974576

[22] Marabelle A, Le DT, Ascierto PA, et al. Efficacy of pembrolizumab in patients with noncolorectal high microsatellite instability/mismatch repair–deficient cancer: results from the phase II KEYNOTE-158 study. J Clin Oncol. 2019;38:1–10 31682550 10.1200/JCO.19.02105 PMC8184060

[23] O'Reilly EM, Oh D-Y, Dhani N, et al. Durvalumab with or without tremelimumab for patients with metastatic pancreatic ductal adenocarcinoma: a phase 2 randomized clinical trial. JAMA Oncol. 2019;5:1431–1438 10.1001/jamaoncol.2019.1588 PMC6647002 31318392

[24] Parikh A, Wo JY-L, Ryan DP, et al. A phase II study of ipilimumab and nivolumab with radiation in metastatic pancreatic adenocarcinoma. J Clin Oncol. 2019;37(4 Suppl):391

[25] Brahmer JR, Tykodi SS, Chow LQM, et al. Safety and activity of anti–PD-L1 antibody in patients with advanced cancer. N Engl J Med. 2012;366:2455–2465 22658128 10.1056/NEJMoa1200694 PMC3563263

[26] Wainberg ZA, Hochster HS, Kim EJ, et al. Open-label, phase I study of nivolumab combined with nab-paclitaxel plus gemcitabine in advanced pancreatic cancer. Clin Cancer Res. 2020;26:4814 LP–4822 LP. 32554514 10.1158/1078-0432.CCR-20-0099

[27] Tran TB, Maker VK, Maker AV. Impact of immunotherapy after resection of pancreatic cancer. J Am Coll Surg. 2019;229:19–27.e1. 30742911 10.1016/j.jamcollsurg.2019.01.016 PMC6599571

[28] The National Cancer Database 2016 PUF Data Dictionary. American College of Surgeons. Available at https://www.facs.org/-/media/files/quality-programs/cancer/ncdb/puf_data_dictionary_2016.ashx Accessed 311, 2021

[29] Lugowska I, Teterycz P, Rutkowski P. Immunotherapy of melanoma. Contemp Oncol. 2018;22:61–67 10.5114/wo.2018.73889 PMC5885078 29628796

[30] Santoni M, Massari F, Di Nunno V, et al. Immunotherapy in renal cell carcinoma: latest evidence and clinical implications. Drugs Context. 2018;7:212528 29899754 10.7573/dic.212528 PMC5992965

[31] Golan T, Hammel P, Reni M, et al. Maintenance olaparib for germline BRCA-mutated metastatic pancreatic cancer. N Engl J Med. 2019;381:317–327 31157963 10.1056/NEJMoa1903387 PMC6810605

[32] Holter S, Borgida A, Dodd A, et al. Germline BRCA mutations in a large clinic-based cohort of patients with pancreatic adenocarcinoma. J Clin Oncol. 2015;33:3124–3129 25940717 10.1200/JCO.2014.59.7401

[33] Wainberg ZA, Hochster HS, Kim EJ, et al. Open-label, phase i study of nivolumab combined with nab-paclitaxel plus gemcitabine in advanced pancreatic cancer. Clin Cancer Res. 2020;26:4814–4822 32554514 10.1158/1078-0432.CCR-20-0099

[34] Zhao P, Li L, Jiang X, et al. Mismatch repair deficiency/microsatellite instability-high as a predictor for anti-PD-1/PD-L1 immunotherapy efficacy. J Hematol Oncol. 2019;12:54 31151482 10.1186/s13045-019-0738-1 PMC6544911

[35] Le DT, Durham JN, Smith KN, et al. Mismatch repair deficiency predicts response of solid tumors to PD-1 blockade. Science. 2017;357:409–413 28596308 10.1126/science.aan6733 PMC5576142

[36] Ahmad-Nielsen SA, Bruun Nielsen MF, Mortensen MB, et al. Frequency of mismatch repair deficiency in pancreatic ductal adenocarcinoma. Pathol Res Pract. 2020;216:152985 32360245 10.1016/j.prp.2020.152985

[37] Vaziri-Gohar A, Zarei M, Brody JR, et al. Metabolic dependencies in pancreatic cancer. Front Oncol. 2018;8:617 30631752 10.3389/fonc.2018.00617 PMC6315177

[38] Bonaventura P, Shekarian T, Alcazer V, et al. Cold tumors: a therapeutic challenge for immunotherapy. Front Immunol. 2019;10:168 30800125 10.3389/fimmu.2019.00168 PMC6376112

[39] Katayama E, Hue JJ, Bajor DL, et al. A comprehensive analysis of clinical trials in pancreatic cancer: what is coming down the pike? Oncotarget. 2020;11:3489–3501 33014285 10.18632/oncotarget.27727 PMC7517959

[40] O'Keefe EB, Meltzer JP, Bethea TN. Health disparities and cancer: racial disparities in cancer mortality in the United States, 2000–2010. Front Public Health. 2015;3:51 25932459 10.3389/fpubh.2015.00051 PMC4398881

[41] Ward E, Jemal A, Cokkinides V, et al. Cancer disparities by race/ethnicity and socioeconomic status. CA Cancer J Clin. 2004;54:78–93 15061598 10.3322/canjclin.54.2.78

[42] Kagawa-Singer M, Valdez Dadia A, Yu MC, et al. Cancer, culture, and health disparities: time to chart a new course? CA Cancer J Clin. 2010;60:12–39 20097836 10.3322/caac.20051

[43] Chin MH, Walters AE, Cook SC, et al. Interventions to reduce racial and ethnic disparities in health care. Med Care Res Rev. 2007;64(5 Suppl):7–28 10.1177/1077558707305413 PMC2366039 17881624

[44] Moy B, Polite BN, Halpern MT, et al. American Society of Clinical Oncology policy statement: opportunities in the patient protection and affordable care act to reduce cancer care disparities. J Clin Oncol. 2011;29:3816–3824 21810680 10.1200/JCO.2011.35.8903

[45] Puts MTE, Monette J, Girre V, et al. Characteristics of older newly diagnosed cancer patients refusing cancer treatments. Support Care Cancer. 2010;18:969–974 20419496 10.1007/s00520-010-0883-0

[46] Restrepo DJ, Sisti A, Boczar D, et al. Characteristics of breast cancer patients who refuse surgery. Anticancer Res. 2019;39:4941–4945 31519599 10.21873/anticanres.13682

